# Improved survival for non-Hodgkin lymphoma patients in New South Wales, Australia

**DOI:** 10.1186/1471-2407-10-231

**Published:** 2010-05-24

**Authors:** Xue Q Yu, Wendy H Chen, Dianne L O'Connell

**Affiliations:** 1 Cancer Epidemiology Research Unit, Cancer Council New South Wales, 153 Dowling Street, Woolloomooloo, NSW 2011, Australia; 2 InforMH, Mental Health and Drug and Alcohol Office, Macquarie Hospital, North Ryde NSW 2113, Australia

## Abstract

**Background:**

We evaluated if the survival benefit of adding rituximab to standard chemotherapy for non-Hodgkin lymphoma (NHL) observed in clinical trials has been experienced by an Australian NHL patient population.

**Methods:**

NHL cases diagnosed in 1985-2004 in New South Wales (NSW) were followed-up to the end of 2004. Rituximab prescription data were obtained from Medicare Australia. Using a Poisson regression model adjusted for age group, sex, NHL subtype and time period (1990-1994, 1995-1999 and 2000-2004), we estimated excess risk of death after a diagnosis of NHL. To give context to the survival trend, trends in incidence and mortality were also estimated.

**Results:**

Compared with 1990-1994, after adjusting for age, sex and NHL subtype the relative excess risk of death was significantly lower (p < 0.0001) in 1995-1999 (0.89) and 2000-2004 (0.74). A sharp fall in mortality was observed from 2000 to 2004 (annual percentage change (APC) = -4.7, p = 0.009), while a small but significant rise in incidence was seen from 1990 to 2004 (APC = 0.5, p = 0.01). The number of times rituximab was dispensed in NSW increased rapidly from 1274 in 1999 to 9250 in 2004.

**Conclusion:**

It is likely that some benefit of adding rituximab to the standard chemotherapy for NHL has been experienced at the population level.

## Background

The incidence of non-Hodgkin lymphoma (NHL) had increased substantially in recent decades with smaller increases in recent years in many western countries including Australia. The mortality for NHL rose at a similar rate, stabilised in the early 1990s and then started to fall at the end of the 1990s. The survival pattern for NHL had not changed significantly in over two decades up to the late 1990s [1] despite attempts to increase the efficacy of the standard treatment, combination chemotherapy (CHOP), with the addition of other cytotoxic drugs [2]. Two pivotal clinical trials in the late 1990s showed that the addition of the monoclonal antibody targeting the CD20 antigen expressed on almost all malignant B cells, rituximab, to standard chemotherapy regimens improved the survival for patients with indolent NHL, and patients with diffuse large B-cell lymphoma (the two commonest NHL subtypes) [1].

This revolutionary advance in the treatment of NHL was introduced in Australia in around 2000 [3,4]. The aim of this study was to evaluate if the introduction of this new treatment modality improved the prognosis of NHL patients in the State of New South Wales (NSW), Australia.

## Methods

### Data

Data were obtained from the population-based NSW Central Cancer Registry, Australia, for cases diagnosed with a single first primary NHL between 1985 and 2004. Based on population size, NSW is the largest state comprising approximately one-third of the Australian population. Notification of cancer has been a statutory requirement for all NSW public and private hospitals, radiotherapy departments and nursing homes since 1972, and for pathology departments since 1985 [5]. Coding for primary site and NHL subtype was done either by medical coders in the hospitals that notified the Registry, or by medical coders in the Registry, who generally assigned subtype based on pathology and hospital notifications. The proportion of cases that were histologically verified has been relatively constant (>85%) since 1985 [5]. Cases aged less than 15 years old, or those reported to the Registry through death certificate only or first identified at post-mortem were excluded. All eligible cases were matched to death records from the State Registrar of Births, Deaths and Marriages and the National Death Index to determine survival status at 31 December 2004.

We obtained the Pharmaceutical Benefits claims data representing the number of times rituximab was dispensed including original prescriptions and repeats [6] in NSW for 1999-2004 from the Medicare Australia website. The prescription codes for NHL patients only were used (8293L, 8294M, 8665C and 8666D), so any increase in the number of claims is directly related to an increase in use for the treatment of NHL. As rituximab was listed for subsidisation in mid 1998, we did not include claims data for that year.

### Trends in survival

Relative survival, a means of removing the effect of mortality from other causes [7], was used in this study because causes of death on death certificates are often inaccurate [8]. Relative survival is the ratio of the observed proportion surviving in a group of patients to the expected proportion that would have survived in a comparable group of people (with the same distribution by age and sex) from the general population [9].

Observed survival was estimated by the life table method [10]. The estimates of the expected survival proportions for each time period (1990-94, 1995-99 and 2000-2004) were derived from the life tables for the general population based on all-cause mortality data and the NSW population by (five-year) age group, sex and calendar year obtained from the Australian Bureau of Statistics. The period method [11] was used to estimate five-year relative survival for the time periods 1990-1994, 1995-1999, and 2000-2004 using the implementation suggested by Paul Dickman [12]. Period analysis applies left truncation to retain the survival experience seen in the period of interest only [11] (in this case, 1990-94, 1995-99 and 2000-2004).

As treatments for NHL depend on histological subtypes [13] and there are approximately 40 different subtypes [14], we grouped all subtypes into four broad groups according to the cell of origin as well as their response to therapy and overall survival. They are "aggressive B-cell", "indolent B-cell", "other NHL (mainly T/NK cells)" and "NHL not otherwise specified (NOS)". The clinical groups were based on the WHO classification for lymphoma [15] and the International Classification of Disease-Oncology Third Edition (ICD-O-3) [16]. Cases diagnosed in 1985-2001 were coded according to ICD-O-2 [17] in the registry; a computer program converted the ICD-O-2 morphology codes into ICD-O-3 morphology codes using a trans-coding table [16]. Cases diagnosed after 2001 were coded in ICD-O-3 in the registry.

Changes in survival over time may be due to several factors, so we assessed the effect of period of survival using multivariable analysis to adjust for confounding variables [18]. More detailed statistical analysis methods for relative survival modeling can be found on Paul Dickman's website http://www.pauldickman.com/. Briefly, we estimated the relative excess risk of death (RER) due to cancer for the 3 periods with 1990-1994 being the reference period, assuming a Poisson distribution for excess deaths [19,20]. In the Poisson model, the dependent variable was the number of excess deaths (calculated as the observed number of deaths minus the expected number of deaths based on the population death rates) with explanatory variables being period of survival, age group at diagnosis (15-44, 45-59, 60-74 and 75+ years), sex, year of follow-up and subtypes of NHL, and the natural logarithm of the population size as the offset. All explanatory variables included in the model were categorical. The RER derived from this model is the ratio of the excess risk of death in a given period to the reference period of 1990-1994 after controlling for the other factors included in the model. A RER smaller than one for a given period indicated that the risk in that period was lower than that for 1990-1994 and vice versa. Ninety-five percent confidence intervals (CIs) for the RERs were calculated using the estimated coefficients and standard errors from the Poisson model. A two-sided, log-likelihood ratio test with p < 0.05 was taken to indicate statistical significance.

All analyses were conducted using SAS version 9.0, and the procedure GENMOD was used to fit the models and assess the prognostic effects of the variables on relative survival.

### Trends in incidence and mortality rates

To estimate trends in incidence and mortality for NHL, which we report as context for the survival trend, annual age-sex standardised incidence and mortality rates were calculated for the NSW resident population for 1990 to 2004. These rates were expressed per 100,000 of the population and age and sex adjusted by the direct method to the 2001 Australian standard population.

Joinpoint regression analysis [21] was used to identify points where a distinct change in the direction or steepness of the trends occurred. The software [22] takes trend data and fits the simplest joinpoint model that the data allows. The analysis starts with the minimum number of joinpoints and identifies any distinct change in direction in the rates and then tests whether the changes are statistically significant. To describe linear trends by period, an annual percent change (APC) is computed for each of those trends.

## Results

A total of 18,798 NHL cases were diagnosed in 1990-2004, with the commonest subtypes being diffuse large B-cell lymphomas (DLBCL) (5022) followed by small lymphocytic lymphoma (SLL)/chronic lymphocytic leukemia (CLL) (3929). Table 1 shows the NHL subtypes and main groups and their ICD-O-3 codes.

**Table 1:** Number of cases by subtype of non-Hodgkin lymphoma with groups according to the WHO classification, 1990-2004 NSW, Australia.

Subtypes and groups	ICD-O-3 codes	No. of cases
**Aggressive B-cell lymphomas**		
Follicular lymphoma, grade 3	9698	179
**Malignant **lymphomas **(ML), mixed small & large cell, diffuse**	**9675**	**466**
Diffuse large B-cell lymphoma	9678-9680, 9684	5022
**Burkitt's lymphoma**	**9687, 9826**	**179**
Lymphoblastic lymphoma	9728, 9836	11
**Indolent B-cell lymphomas**		
Follicular lymphomas (grade 1 and 2)	9690-9691, 9695	2558
**Marginal zone lymphoma of mucosa-associated lymphoid tissue (MALT)**	**9689, 9699**	**302**
Small lymphocytic lymphoma, Chronic lymphocytic leukemia	9670, 9823	3929
**Hairy cell leukemia**	**9940**	**277**
Lymphoplamacytic lymphomas	9671, 9761	472
**Other NHL**		
Mature T-cell lymphomas: peripheral, angioimmunoblastic, hepatosplenic gamma/delta T-cell	9702, 9705, 9708, 9714, 9716, 9827	245
**Cutaneous T-cell**	**9709**	**85**
Mycosis fungoides and mantle cell	9700-9701, 9673	422
**Other T/NK cell lymphomas**	**9717, 9718, 9719, 9831, 9948**	**58**
Precursor T-cell	9729, 9837	11
**NHL not otherwise specified (NOS)**		
ML, NOS	9590	1191
**ML, non-Hodgkin, NOS**	**9591**	**2701**
Precursor cell lymphoblastic lymphoma, NOS	9727	72
**Lymphoid leukemia, NOS**	**9820**	**566**
Immunoproliferative disease, NOS	9760	32
**Precursor cell lymphoblastic leukemia NOS**	**9835**	**20**

Table 2 shows the characteristics of the NHL patients by calendar period.

**Table 2:** Characteristics of patients with non-Hodgkin lymphoma, NSW Australia, 1990-2004.

	Number of cases	Percentage (%)		
		
		1990-1994 (n = 5454)	1995-1999 (n = 6231)	2000-2004 (n = 7113)
**Sex**				
Males	10,475	56.3	55.2	55.7
Females	8,323	43.7	44.8	44.3
**Age at diagnosis (years)**				
15-44	2,484	15.2	13.2	11.7
45-59	4,110	20.5	21.8	22.9
60-74	6,905	38.0	37.1	35.4
75-89	5,299	26.3	27.9	29.9
**NHL groupings**				
Aggressive B-cell	5,857	32.4	33.3	28.3
Indolent B-cell	7,538	39.5	38.2	42.2
Other NHL	821	1.4	4.1	6.9
NHL NOS	4,582	26.6	24.5	22.5

There was a substantial increase in 5-year relative survival for patients with NHL from 1990-1994 to 2000-2004 (53.3% vs 62.1%) (Table 3). Compared with the survival experience of patients in 1990-94, relative excess risk of death was significantly lower (p < 0.0001) in 1995-99 (RER = 0.89, 95% CI: 0.84-0.94) and 2000-2004 (RER = 0.74, 95% CI: 0.69-0.78) after adjusting for age, sex and NHL subtype. Table 3 also shows the estimated 5-year relative survival and relative excess risk of death for levels of the other covariates included in the Poisson model.

**Table 3:** Five-year relative survival (RSR) and relative excess risk (RER) during the first five years after diagnosis of NHL, NSW Australia 1990-2004.

	5-yr RSR (%)	RER*	95% CI†	P-value
**Period of survival**				<0.0001
1990-1994	53.3	1.00		
1995-1999	55.9	0.89	(0.84-0.94)	
2000-2004	62.1	0.74	(0.69-0.78)	
**Sex**				<0.0001
Males	57.5	1.00		
Females	57.5	0.86	(0.82-0.91)	
**Age at diagnosis (years)**				<0.0001
15-44	66.6	1.00		
45-59	70.3	1.03	(0.94-1.13)	
60-74	58.3	1.56	(1.44-1.70)	
75-89	39.5	2.95	(2.71-3.20)	
**NHL grouping**				<0.0001
Agressive B-cell	45.2	1.00		
Indolent B-cell	73.1	0.30	(0.28-0.32)	
Other NHL	61.0	0.92	(0.86-0.97)	
NHL NOS	46.6	0.62	(0.54-0.71)	

* Adjusted for all covariates shown in the table using a Poisson regression model

† CI - confidence interval

**Figure 1:**
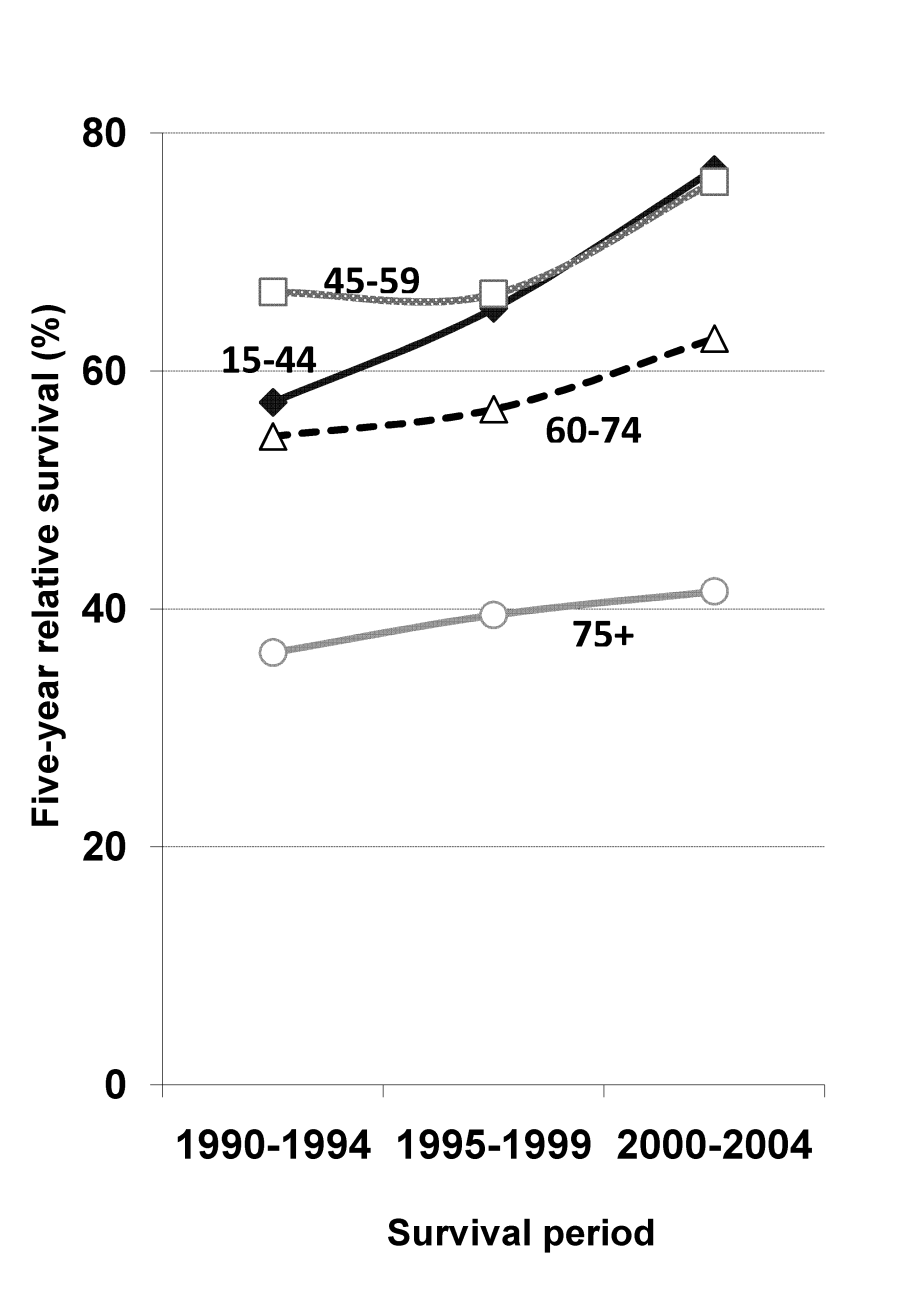
**Trends in five-year relative survival for NHL by age group at diagnosis, NSW Australia, 1990-2004**.

Five-year relative survival in 2000-2004 was higher for all age groups with those very old (75+ years) having poorer outcomes (Figure 1).

**Figure 2:**
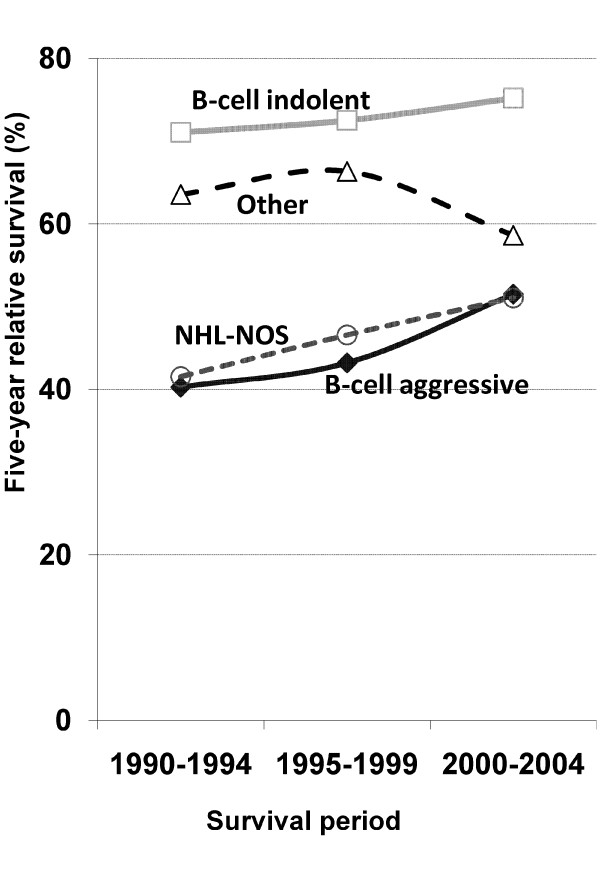
**Trends in five-year relative survival for NHL by histological subtype, NSW Australia, 1990-2004**.

Five-year relative survival in 2000-2004 was higher for all subtypes except for 'Other NHL' (Figure 2).

**Figure 3:**
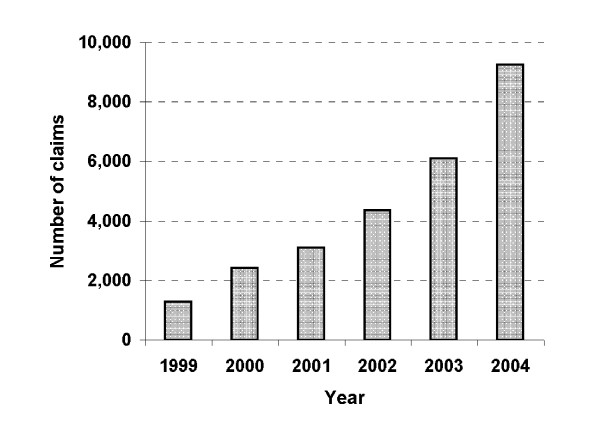
**Number of times rituximab was dispensed for treatment of NHL, NSW Australia, 1999-2004**.

The number of times rituximab was dispensed for the treatment of NHL increased rapidly from 1999 (1274) to 2004 (9250) in NSW (Figure 3).

The trends in mortality suggested that a small rise from 1990 to 1996 (APC = 0.8, p = 0.44) was followed by a substantial but non-significant fall (APC = -2.1, p = 0.41) from 1996 to 2000 and then a further sharp fall from 2000 to 2004 (APC = -4.7, p = 0.009). There was a small but significant increase in incidence from 1990 to 2004 (APC = 0.5, p = 0.02).

## Discussion

In this population-based study, we found that a substantial improvement in survival for NHL (5-year relative survival 62% in 2000-2004 vs 53% in 1990-1994) coincided with a sharp fall in mortality after 2000. This was approximately when rituximab was approved by the Australian Therapeutic Goods Administration for clinical use in Australia for the two commonest NHL subtypes, namely low grade or follicular lymphoma (June 1998) [3] and diffuse large B-cell lymphomas (February 2002) [4]. These results are consistent with the beneficial effects of rituximab on cancer survival observed in clinical trials [23-25].

The findings of this population-based study are important because it includes all patients with NHL, many of whom would not have met the trials' inclusion criteria, in a well-defined geographical area, thus reflecting the experience of real world everyday clinical practice. The main strength of the study was that we grouped subtypes of lymphomas into treatment-based categories classified according to WHO guidelines. This grouping has been used by others [26], is clinically useful [27,28] and reduces the probability of misclassification bias [28]. Another strength is that we studied the trends in the incidence of, survival for and mortality from NHL simultaneously, and interpreted the survival trend in the context of incidence and mortality trends, as recommended by several authors [29-31].

What may explain the better survival observed in the most recent period in this study? Several factors may have contributed to the improved survival in 2000-2004. First, high-dose chemotherapy with shorter intervals between treatment cycles [32], together with better supportive care including antibiotics and growth factor to manage myelotoxicity [33], was introduced during the study period. An Australian study has also demonstrated that growth factor can be used safely and efficaciously to support intensified chemotherapy (CHOP-14) in Australian NHL patients [34]. However it only became available in 2003 and it was dispensed 1560 times in NSW in 2004 [6] so is unlikely to explain the improved survival in 2000-2004. Second, more NHL patients in NSW may have participated in clinical trials [35] which may in part have led to improved survival in the later period. Third, the introduction of highly active antiretroviral therapy in Australia around 1997 [36] may explain in part the improved survival in 2000-2004 [37]. The substantial (although not significant) decline (-2.1% per year) in mortality from 1996 to 2000 in our data provides further support for this. Fourth, changes in case-mix over time may have accounted for some of the survival benefits observed in 2000-2004. However our use of four broad categories of lymphoma subtype should reduce this problem to a minimum [28]. As shown in Table 2 the case-mix in different time periods was generally comparable except for "Other NHL".

Finally, increasing survival over time may reflect improvements in earlier detection by screening programs. However, there is no possibility for early detection of NHL. Indeed, a small and gradual increase in incidence was seen from 1990 to 2004, not a sudden substantial increase in the 2000-2004 period. Therefore, the coincidental sharp fall in mortality from 2000 to 2004 indicates that these improvements in outcomes were due to effective therapy [30,31,38]. Indeed, the therapeutic monoclonal antibody, rituximab, was introduced into clinical use in Australia initially for patients with relapsed or refractory indolent B-cell NHL (follicular or small lymphocytic subtypes) in 1998, and then for patients with diffuse large B-cell lymphoma (the commonest type of aggressive NHL) in early 2002. The use of rituximab for treating B-cell NHL has been wide spread in Australia (Figure 3) as universal health coverage is available and, consistent with findings from clinical trials, the improved survival was mainly seen in B-cell lymphomas and was of a similar magnitude to that reported in the trials [23,39]. It is likely that these 5-year relative survival estimates in 2000-2004 derived by period analysis underestimate the true later observed relative survival [40] given the rapid increase in the use of rituximab for treatment of NHL in Australia. Taking all this into account, we are reasonably confident that the introduction of rituximab is a major contributor to the improved survival observed in 2000-2004.

Our results are also consistent with other population-based studies examining trends in NHL survival over time [41,42]. In a recent study using data from the Surveillance, Epidemiology, and End Results (SEER) program, Pulte et al found that 5-year relative survival for NHL patients in the United States improved significantly between 1990-92 and 2002-2004 (from 50.4% to 66.8%) [41]. The apparent greater improvement in survival observed by Pulte et al [41] over the same time period as our study may be explained by the shorter time intervals used for their analysis (three years, not five years) with the greatest benefits from the use of rituximab occurring in the most recent period. However, the overall consistency of these results with those we reported during an almost identical study period provides indirect confirmation of our findings. The improved survival in 2000-2004 we observed was also comparable to that of a population-based study in Canada [42]. However, the magnitude of the survival improvement that we observed was more modest. This may be due to the fact that they excluded patients with poor prognosis such as those who were HIV positive and those who did not receive curative treatment [42] whereas our study included all patients with aggressive NHL.

Age at diagnosis was inversely associated with survival (Figure 1) which is probably due to more co-morbidities and adverse effects of chemotherapy in older patients. However, the improved survival over time was seen for both older and younger patients (Figure 1), although it was less for those very old (75+ years). This is consistent with evidence from clinical trials: survival increased for both older (60-80 years) [23] and younger (18-60 years) patients [35] after the addition of rituximab to standard chemotherapy for aggressive B-cell lymphomas, the commonest NHL subtype.

The relative survival trends for NHL NOS and aggressive B-cell lymphomas were similar (Figure 2). This suggests that the former group of patients also received rituximab because previous studies showed that patients who did not receive cancer-related therapy had poorer outcomes [43,44]. Improved diagnostic specificity during the 1990s [45] did not decrease the proportion of NOS lymphomas over time (Table 2). This is very similar to the results of a previous study where a SEER registry was reviewed by an expert hematopathologist (24.3% vs 25%) [46] and suggests that it may not be possible to decrease this proportion further on a population-based cancer registry.

The data used in this study have some limitations. The cancer registry does not collect detailed treatment information and so the data do not allow us to directly conclude that the survival benefit observed over time was due to the use of rituximab in the treatment of NHL, although the Pharmaceutical Benefits claims data (Figure 3) provided indirect support for the observed improved survival in 2000-2004. However, these data do not allow us to estimate the fraction of patients diagnosed in 2000-2004 who actually used rituximab, thus the possibility that some unmeasured factors mentioned earlier might also have contributed to this improved survival cannot be ruled out completely. Another limitation was that the cancer registry does not collect information on patients' comorbidities and disease extent for NHL. These factors are related to the prognosis for NHL [44]. However, our use of relative survival to account for competing mortality addressed these factors at least in part.

## Conclusions

The observed rise in survival together with a fall in mortality from 2000 and a rapid increase in the use of rituximab, suggests that the benefit of adding rituximab to the standard treatment of NHL may have been experienced at least in part by this Australian NHL patient population. There are good grounds for believing that further improvement in survival, especially for patients with aggressive B-cell lymphomas, will almost certainly continue to increase as more follow-up time accrues.

## Competing interests

The authors declare that they have no competing interests.

## Authors' contributions

XQY designed the study, obtained the cancer registry data, provided oversight of the data analysis, and drafted the manuscript. WHC participated in the design of the study, performed the data analysis, and helped to draft the Methods and Results sections. DLO participated in the study design, obtained the prescription data, and revised the manuscript critically. All authors read and approved the final version of the manuscript.

## Pre-publication history

The pre-publication history for this paper can be accessed here:

http://www.biomedcentral.com/1471-2407/10/231/prepub
